# Absence of the common *Insulin-like growth factor-1* 19-repeat allele is associated with early age at breast cancer diagnosis in multiparous women

**DOI:** 10.1038/sj.bjc.6603632

**Published:** 2007-02-20

**Authors:** E Bågeman, C Ingvar, C Rose, H Jernström

**Affiliations:** 1 Department of Oncology, Clinical Sciences, Lund University, SE-221 85 Lund, Sweden; 2 Department of Surgery, Lund University Hospital, SE-221 85 Lund, Sweden; 3 Department of Oncology, Lund University Hospital, SE-221 85 Lund, Sweden

**Keywords:** *IGF1* genotype, multiparity, early-onset breast cancer

## Abstract

Multiparity decreases the risk of breast cancer in white women, whereas it is a risk factor in black women <50 years. Early-onset breast cancer (<50 years) has been associated with high insulin-like growth factor-1 (IGF-1) levels. Absence of the common *IGF1* 19 cytosine-adenine (CA)-repeat allele (*IGF1-19/-19*) inverts the effect of several non-genetic factors on breast cancer risk but the interaction between *IGF1-19/-19* and multiparity on breast cancer risk is unknown. As *IGF1-19/-19*, multiparity and early-onset breast cancer are more common in black than in white women, we aimed to study whether multiparity combined with *IGF1-19/-19* increases the risk of early-onset breast cancer. Four hundred and three breast cancer patients diagnosed in Lund, Sweden, at age 25–99 years were genotyped for the *IGF1* CA-repeat length using fragment analysis. Overall, 12.9% carried the *IGF1-19/-19* genotype. There was a highly significant interaction between multiparity and *IGF1-19/-19* on age at breast cancer diagnosis (*P*=0.007). Among *IGF1-19/-19* patients, multiparity was associated with a 9.2 year earlier age at diagnosis compared with uniparity or nulliparity (*P*=0.006). Multiparity combined with *IGF1-19/-19* was associated with an early age at breast cancer diagnosis. If confirmed, *IGF1-19/-19* may help identify a subgroup of women for earlier breast cancer screening.

Breast cancer is the most common malignancy in women worldwide (Parkin *et al*, 2001, 2002). Approximately 10% of women living in Sweden will develop the disease during their lifetime. The median age at breast cancer diagnosis in women in Sweden is 60 years; approximately 4% of women are 40 years or younger at diagnosis (data from the Swedish Cancer Registry). The prognosis is often poorer in younger than in older women (Sidoni *et al*, 2003) in terms of overall survival and relapse (Han *et al*, 2004), partly because young women do not benefit from breast cancer screening and early-onset breast cancers are usually detected clinically at a more advanced stage. In Lund, Sweden, mammography screening is initiated at age 45 years and is performed every 18 months until the age of 74 years. As of today, no tests are routinely performed to identify the subgroup of women who do not belong to high-risk breast cancer families (eg *BRCA1* families) but who would nevertheless benefit from breast cancer screening at an earlier age owing to an increased risk for early-onset breast cancer.

Breast cancer risk is determined by genetic factors and non-genetic factors, for example, reproductive factors (Beral and Reeves, 1993; Kelsey *et al*, 1993). An increased age at first full-term pregnancy and low parity have been associated with an increased risk of developing breast cancer in the developed world (Kelsey *et al*, 1993). In the short term, a first full-term pregnancy increases the risk of breast cancer (Beral and Reeves, 1993). Each subsequent live-birth reportedly decreases the risk of breast cancer by 30% and after three full-term pregnancies, the short-term risk of developing breast cancer is similar to the risk of nulliparous women (McCredie *et al*, 1998). In the long-term, a full-term pregnancy confers a decreased risk of developing breast cancer (McCredie *et al*, 1998) and each subsequent full-term pregnancy magnifies this protective effect.

In the developing world, a higher proportion of the diagnosed breast cancers are early-onset breast cancers Parkin *et al* (2002) compared to the developed world. This fact is unexpected, as multiple pregnancies and young age at first full-term pregnancy are much more common in the developing world than in the developed world. Therefore, the higher proportion of early-onset breast cancer may be owing to either genetic or non-genetic factors aside from reproductive factors. That genetic factors play an important role is suggested by studies, which show that early-onset breast cancer is more common in African-American women than in white women in the US (Amend *et al*, 2006; Ries *et al*, 2006) and that multiparity is associated with an increased breast cancer risk before age 45 years in young African-American women (Palmer *et al*, 2003), but not in young white women (Hall *et al*, 2005).

Insulin-like growth factor-1 (IGF-1) levels are higher in young black woman than in young white women (Jernström *et al*, 2001a) and a higher level of circulating IGF-1 is a known risk factor for early-onset breast cancer (Peyrat *et al*, 1993; Hankinson *et al*, 1998; Renehan *et al*, 2004; Sugumar *et al*, 2004; Fletcher *et al*, 2005; Schernhammer *et al*, 2005). It has been estimated that between 38 and 77% of the individual variation in IGF-1 levels is because of genetic factors (Harrela *et al*, 1996; Verhaeghe *et al*, 1996). Circulating IGF-1 levels may be modified (Rosen *et al*, 1998) by a cytosine-adenine (CA) repeat in the proximity of the promoter, 1 kb upstream from the transcription start site of the *IGF1* gene (Rotwein *et al*, 1986). The 19-repeat allele is the most common CA repeat length among white women and only 6–13% of white women have no 19-repeat allele (*IGF1-19/-19* genotype) (Jernström *et al*, 2001a, 2001b, 2005; Vaessen *et al*, 2001; DeLellis *et al*, 2003), whereas more than 32% of black women and men have the *IGF1-19/-19* genotype (Jernström *et al*, 2001a; Schildkraut *et al*, 2005). The *IGF1-19/-19* genotype is thus approximately three times more common in black women than in white women. Furthermore, the *IGF1-19/-19* genotype is present in 16% of Indian-Pakistani women and in over 33% of other Asian women (Jernström *et al*, 2001a; Wen *et al*, 2005).

Breast cancer risk appears to be influenced by the interaction between the *IGF1* genotype and non-genetic factors. Oral contraceptive (OC) use in combination with the *IGF-19/-19* genotype increases the risk of early-onset breast cancer (Jernström *et al*, 2005; Cleveland *et al*, 2006). In women who never had used OCs, the *IGF1-19/-19* genotype was associated with a decreased risk of early-onset breast cancer as compared with women who have at least one copy of the 19-repeat allele (*IGF1*+*19*) (Cleveland *et al*, 2006). Oral contraceptive use decreased IGF-1 levels in women with the *IGF1*+*19* genotype but increased IGF-1 levels in white, black, Asian and Indian-Pakistani women with the *IGF-19/-19* genotype (Jernström *et al*, 2001a). The effects of hormone replacement therapy (HRT), alcohol intake, body mass index (BMI) and smoking on breast cancer risk were all likewise modified by the presence or absence of the *IGF-19/-19* genotype (Cleveland *et al*, 2006). The interaction between multiparity and the *IGF1-19/-19* genotype on breast cancer risk was not addressed in the study by Cleveland *et al*, 2006.

The protective effect of increasing parity on breast cancer risk in the developed world is thought to be mediated in part by decreasing circulating IGF-1 levels (Holmes *et al*, 2002). As multiparity is protective against breast cancer in the developed world but not in the developing world, as the *IGF1-19/-19* genotype is more common among black, Indian-Pakistani and other Asian women than among white women, and as the *IGF1-19/-19* genotype inverts the effect of several non-genetic factors on breast cancer risk, we aimed to investigate whether the effect of multiparity on age at breast cancer diagnosis is influenced by the *IGF1-19/-19* genotype.

## MATERIALS AND METHODS

All women with a primary breast cancer were invited to take part in an ongoing study of breast cancer at the Lund University Hospital, Sweden, regardless of ethnic background, age and stage. Patients who had recently been diagnosed and treated for another type of cancer were not eligible to participate. Four hundred and three patients were included between October 2002 and July 2006. The study was approved by the Ethics Committee of the Lund University. All patients provided a written informed consent.

During the pre-operative visit to the Department of Surgery of the Lund University Hospital, a research nurse collected blood samples (EDTA plasma and serum) and recorded the time and date when the blood samples were drawn. The blood was centrifuged and separated. Serum, EDTA plasma and blood cells were stored at −70°C. All samples were labelled with serial codes to enable blinded analyses.

Body weight, height, waist and hip circumferences and breast volumes were measured. The breast volume was measured with plastic cups used by plastic surgeons doing breast reductions. They come in the following 11 sizes: 200, 275, 350, 500, 650, 800, 950, 1150, 1325, 1500 and 2000 ml (Ringberg *et al*, 2006).

All patients filled out a baseline questionnaire including questions on the use of exogenous hormones and concomitant medications, smoking, alcohol intake, reproductive history and family history of cancer.

Patients who had not experienced a menstrual period during the last year were defined as postmenopausal. However, postmenopausal patients who used HRT may have had HRT induced bleeding and may therefore have been misclassified as premenopausal. Patients who had had their uterus removed before menopause but not their ovaries may also have been misclassified as postmenopausal. We therefore classified patients according to age (< 50 years or ⩾50 years of age) instead of reported menopausal status. We chose the cutoff at age 50 years as others have shown that high IGF-1 levels influence the risk of breast cancer in women under the age of 50 years (Hankinson *et al*, 1998). We also chose to use a cutoff age of 45 years as Lund's screening program for breast cancer starts at this age.

Additional baseline information including type of surgery, sentinel node biopsy, axillary node dissection, tumour size, histological type and grade, axillary node involvement, signs of distant metastases, oestrogen receptor and progesterone receptor status was obtained from each patient's chart and pathology report. Oestrogen receptor status and progesterone receptor status were reported as either positive or negative. HER-2/neu status was not routinely analysed.

### Genetic analysis

Genomic DNA was extracted from 300 *μ*l of peripheral blood using Wizard, Genomic DNA Purification Kit (Promega, Madison, WI, USA). The CA repeat in the proximity of the *IGF1* promoter ranged in size from 11 to 23 repeats. Polymerase chain reaction (PCR) primers 5′-GCTAGCCAGCTGGTGTTATT and 5′-GTTTCTTACCACTCTGGGAGAAGGGTA were used, where the forward primer was fluorescently labelled with FAM (MWG-Biotech AG, Ebersberg, Germany). Polymerase chain reaction was performed in 15 *μ*l reactions using 25 ng of DNA, 0.4 *μ*M of each primer, 0.1 *μ*M of each deoxynucleotide (Amersham Biosciences, Buckinghamshire, UK), 5% DMSO (Sigma, St Louis, MO, USA), 2.5 mM MgCl_2_ (Applied Biosystems, Foster City, CA, USA), 1 × PCR Gold Buffer (Applied Biosystems, Foster City, CA, USA) and 1 U AmpliTaq Gold (Applied Biosystems, Foster City, CA, USA).

The PCR product was analysed in an ABI3100 Prism Genetic Analyzer (Applied Biosystems, Foster City, CA, USA) and the results were evaluated using Genescan software. The number of repeats was determined by sequencing samples of varying sizes (Big Dye, Terminator Cycle Sequencing, Applied Biosystems, Foster City, CA, USA) and using them as standards in the fragment analysis. The results from the ABI3100 Prism Genetic Analyzer were manually evaluated and each sample was read in duplicate. The first 360 samples were analysed using this method.

For quality control, the first 352 samples were run in duplicate in separate PCR and fragment analyses. Thereafter, every fourth sample was run in duplicate. The concordance rate was 98.3%. The discordant samples were re-checked, and we found that the genotypes were identical in the separate PCR and fragment analyses, but that the wrong peaks had been manually selected during one of the readings.

In April 2006, the system was upgraded to an ABI Prism 3130*xl* Genetic Analyzer (Applied Biosystems, Foster City, CA, USA). Forty-three samples were run on the upgraded system. The repeat lengths were manually evaluated, as stated above, as well as automatically evaluated by the GeneMapper Software v.4.0. The concordance rate was 100% between the manual and automatic readings. The GeneMapper software could not determine repeat sizes below 17 repeats, but these alleles are rare (0.2%).

### Statistical analysis

Data analysis was performed with the statistical software SPSS 13.0. for Windows (SPSS Inc. Chicago, IL, USA). For univariate analyses, *χ*^2^ square analysis was used for dichotomous variables and Student's *t*-test was used for continuous variables. Age at diagnosis was used as a continuous variable. Age at diagnosis was also categorised as age < 50 years (yes/no) and age < 45 years (yes/no). Multiparity was classified as having two or more children (yes/no). The *IGF1-19/-19* genotype was classified as having no *IGF1* 19-repeat allele (yes/no). Multivariate linear regression was used to examine the relationship between the *IGF1-19/-19* genotype and multiparity on age at breast cancer diagnosis. An interaction variable was computed between these two factors. Adjustments were carried out for other factors such as OC use, HRT use, alcohol intake, BMI and current smoking. The study was based on an *a priori* hypothesis, namely, that the *IGF1* genotype in combination with multiparity, is associated with early-onset breast cancer. Therefore no adjustment for multiple testing is required for the *P*-value for interaction, neither in the univariate nor in the multivariate analyses. All *P*-values are two-tailed and presented without Bonferroni correction for multiple testing.

## RESULTS

### Age at diagnosis and parity

The baseline characteristics for all patients (*n*=403) are shown in Table 1. Age at breast cancer diagnosis ranged from 25 to 99 years, with a mean age of 58.5 years. We classified patients diagnosed < 50 years of age as younger patients (*n*=96) and patients diagnosed ⩾50 years of age as older patients (*n*=307). One hundred and twenty-eight patients were nulliparous or uniparous whereas 275 patients were multiparous, that is, had two or more children. Parity did not differ between the younger and the older patients (1.8 children *vs* 1.9 children; *P*=0.49), but age at first full-term pregnancy was higher in the younger than in the older patients (26.6 *vs* 24.6 years; *P*=0.001, significant also after Bonferroni correction). The only factor included in Table 1 that was associated with the *IGF1-19/-19* genotype was age at breast cancer diagnosis. Patients with the *IGF1-19/-19* genotype had a 3.5-year earlier age at diagnosis compared with patients with the *IGF1*+*19* genotype, (*P*=0.04).

### *IGF1-19/-19* genotype frequency and age at diagnosis

**Figure 1:**
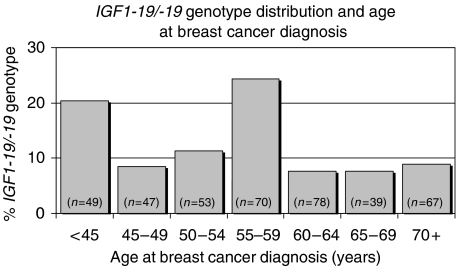
Frequency of the *IGF1-19/-19* genotype in the 403 patients according to age at diagnosis. The total number of women in each age category is indicated. The *IGF1-19/-19* genotype was most common (over 20 %) in patients diagnosed before age 45 years and in patients diagnosed between the ages of 55 and 59 years and thus displayed a biphasic distribution.

The allele frequency distribution of the *IGF1* CA-repeat length in all patients is described in Table 2. Overall, 12.9% carried the *IGF1-19/-19* genotype. The distribution was not equal in all age groups. The *IGF1-19/-19* genotype was most common (over 20%) in patients diagnosed before age 45 years and in patients diagnosed between the ages of 55 and 59 years, and thus displayed a biphasic pattern, (Figure 1). Age did not differ between patients who were heterozygous and patients who were homozygous for the 19-repeat allele (59.0 *vs* 58.8 years; *P*=0.86). We therefore combined these two groups and classified their genotypes as *IGF1*+*19*.

### The frequency of *IGF1-19/-19* genotype in relation to parity and age at diagnosis

Younger patients with the *IGF1-19/-19* genotype had more children than younger patients with the *IGF1*+*19* genotype (2.4 children *vs* 1.7 children; *P*=0.01). In fact, only one young patient with the *IGF1-19/-19* genotype had less than two children, that is, one child. Parity did not differ by *IGF1-19/-19* genotype among the older patients (1.9 children *vs* 1.9 children; *P*=0.85).

Among the younger multiparous patients, 19.7% carried the *IGF1-19/-19* genotype compared with 11.6% among all other patients, odds ratio (OR) 1.7 (95% confidence interval (CI) 0.96–3.0; *P*=0.07). As mammography is initiated at age 45 years in Lund, Sweden, we reanalysed our results using 45 years as an age cutoff. With this age cutoff, the *IGF1-19/-19* genotype was more than twice as common in multiparous patients diagnosed before age 45 than in other patients (27.3% *vs* 11.6%), OR 2.3 (95% CI 1.3–4.4; *P*=0.01).

### The effect of *IGF1-19/-19* genotype and parity on age at diagnosis

In multiparous patients with the *IGF1-19/-19* genotype, the mean age at breast cancer diagnosis was 9.2 years earlier compared with uniparous or nulliparous patients (*P*=0.006). Age at breast cancer diagnosis was not significantly influenced by multiparity in women with the *IGF1*+*19* genotype (*P*=0.33). The interaction between multiparity and the *IGF1-19/-19* genotype on age at breast cancer diagnosis, which was our *a priori* hypothesis, was highly significant (*P*=0.007) (Table 3), and it remained significant after adjustment for ever OC use, ever HRT use, alcohol intake, BMI and current smoking (*P*_interaction_=0.03).

Nine patients had twins. Five of these nine patients had only one full-term pregnancy. The results remained essentially unchanged when these patients were reclassified as uniparous instead of multiparous. We then analysed the effect of recognised pregnancies (whether full-term or not). The interaction between two or more recognised pregnancies and the *IGF1-19/-19* genotype on the age at breast cancer diagnosis was still significant (*P*=0.02). The one uniparous patient with the *IGF1-19/-19* genotype whose breast cancer was diagnosed before age 50 years had in fact had several recognised pregnancies; no patient with the *IGF1-19/-19* genotype with less than two recognised pregnancies was diagnosed before age 57 years.

### *IGF1* genotype and mode of cancer detection, tumour characteristics and family history of breast cancer

Among the 322 patients aged 45–74 years, there was no difference in mode of detection in patients with or without the *IGF1-19/-19* genotype (*P*=0.82). *IGF1* genotype was associated with neither of the following tumour characteristics: tumour size, nodal involvement, grade, oestrogen receptor status or progesterone receptor status in either age group (all *P* values ⩾0.16) nor with a history of breast cancer in a first and/or second degree relative (*P*=0.70).

## DISCUSSION

The main finding of this study was that there was a highly significant interaction between multiparity and the *IGF1-19/-19* genotype on age at breast cancer diagnosis. Multiparous patients with the *IGF1-19/-19* genotype had a 9.2-year earlier age at breast cancer diagnosis compared with nulliparous or uniparous patients with the same genotype. Among our study population, no patient with the *IGF1-19/-19* genotype and less than two recognised pregnancies (whether full-term or not) was diagnosed before age 57 years. Among women with the *IGF1*+*19* genotype, multiparity had no significant effect on age at diagnosis. The interaction between the *IGF1-19/-19* genotype and multiparity on the age at breast cancer diagnosis has to our knowledge not been reported previously.

We also found that the *IGF1-19/-19* genotype was more than twice as common among the youngest patients (diagnosed <45 years of age) who were multiparous compared with all other patients. It was noteworthy that only one of the young breast cancer patients with the *IGF1-19/-19* genotype had less than two children. This observation strengthens the hypothesis that it is the combination of the *IGF1-19/-19* genotype and multiparity that is deleterious and suggests that the *IGF1-19/-19* genotype may be associated with a substantial number of the early-onset breast cancers in the developing world where both this genotype and multiparity are common. Conversely, in China, where it is uncommon to have more than one child, the *IGF1-19/-19* genotype was associated with a decreased risk of early onset breast cancer (Wen *et al*, 2005). Their result is in line with our finding that patients with the *IGF1-19/-19* genotype and fewer than two pregnancies had a later age at diagnosis.

As suggested by Holmes *et al* (2002) parity may exert its protective effect by lowering IGF-1 levels post-partum. Such results were not replicated in a previous study of young white women from high-risk breast cancer families (Jernström *et al*, 2005). In that study, the IGF-1 levels of parous and nulliparous women were similar. Furthermore, we found that the frequency of the *IGF1-19/-19* genotype was significantly higher among known *BRCA1* carriers compared with other high-risk women and we have reported previously that each pregnancy up to three confers an increased risk for breast cancer before age 40 years among *BRCA1* and *BRCA2* carriers (Jernström *et al*, 1999). We therefore hypothesise that the IGF-1 levels do not decrease post-partum in women with the *IGF1-19/-19* genotype. Each pregnancy may thus confer an increased risk of early-onset breast cancer.

In our study, patients with the *IGF1-19/-19* genotype who had two or more children had a lower age at diagnosis. In the study by Cleveland *et al* (2006), the *IGF1-19/-19* genotype modified the breast cancer risk after exposure to hormonal factors such as OCs and women with the *IGF1-19/-19* genotype who had ever used OCs had an increased breast cancer risk whereas this genotype was protective in women without OC exposure. However, our results remained significant after adjustment for OC use, HRT use as well as alcohol intake, BMI and current smoking.

The frequency of the *IGF1-19/-19* genotype was 12.9% in our patient population and is consistent with the frequency among white women that has been reported to vary between 6 and 13% (Vaessen *et al*, 2001; Jernström *et al*, 2001b, 2005; DeLellis *et al*, 2003). In this study, we observed a biphasic variation in the frequency of the *IGF1-19/-19* genotype in relation to age at diagnosis. This observation needs confirmation in other studies, as it has not been previously reported. Among multiparous patients diagnosed before age 50 years, the frequency of the *IGF1-19/-19* genotype was almost twice as common as in the other patients. As mammography screening is initiated at the age of 45 years in Lund, Sweden, we then re-analysed our results. The frequency of the *IGF1-19/-19* genotype in the multiparous patients (< 45 years) was then more than twice as common as in the other patients. As examining the frequency of the *IGF1-19/-19* genotype in relation to different age cutoffs was not part of our *a priori* hypothesis, these results have to be regarded as hypothesis generating. However, they suggest that multiparous patients with the *IGF1-19/-19* may not only have an earlier age at breast cancer diagnosis but also an increased risk of developing the malignancy.

This study was based on a series of breast cancer patients diagnosed with a primary breast cancer at Lund University Hospital, Sweden. All breast cancers were verified by the Department of Pathology, Lund University Hospital. The baseline information was filled out by all patients at the pre-surgical visit, which minimises bias owing to survival differences between patients.

It has been suggested that contradictory findings regarding *IGF1 CA-*repeat lengths and breast cancer risk may be owing to differences in the methods used to determine the repeat lengths, sequencing or fragment analysis (Tran *et al*, 2004). The repeat numbers obtained with fragment analysis have differed when analysing the results on an ABI Prism 3100 Genetic Analyzer using the internal standard supplied by the manufacturer as compared to the results received when using a ladder that has been verified by direct sequencing (Rodriguez *et al*, 2006). It is therefore important to emphasise that all the genetic analyses in this study were performed with the same method, that is, fragment analysis on an ABI Prism 3100 or a 3130*xl* Genetic Analyzer, using control samples with lengths verified by direct sequencing. To evaluate the reproducibility of this method, 353 results were re-run and the concordance rate was 98.3%. When re-examining the discordant samples, we found that the errors were of human nature in misinterpreting the technically sound genotype results. This will not be an issue in the future when the samples will also be automatically evaluated by the GeneMapper software.

The *IGF1* genotype was not associated with TNM staging, grade or hormone receptor status in either age group, nor was it associated with mode of detection among the patients whose cancer was diagnosed between the ages of 45 and 74 years. As early-onset breast cancer in itself is associated with a poorer prognosis (Sidoni *et al*, 2003), it may be advantageous to initiate breast cancer screening among multiparous women with the *IGF1-19/-19* genotype at an earlier age. Alternative screening methods may be needed in this age group as mammography shows poor sensitivity in young women (Houssami *et al*, 2003). Conversely, among patients with fewer than two children, the *IGF1-19/-19* genotype was associated with a later age of breast cancer onset, and these women may initiate screening at a later age. If confirmed, our data suggest that *IGF1* genotyping should be considered in all women with multiple pregnancies to determine the optimal start age for breast cancer screening.

## CONCLUSION

Our findings suggest that the combination of *IGF1-19/-19* genotype and multiparity is associated with an early age at breast cancer diagnosis. These findings may explain why the proportion of early-onset breast cancers is higher in the developing than in the developed world. Further studies are warranted to confirm our findings. If confirmed, the *IGF1-19/-19* genotype may help identify a subgroup of patients for whom early breast cancer screening is warranted as well as a subgroup of patients for whom the onset of regular screening can safely be postponed.

## Figures and Tables

**Table 1:** Baseline characteristics of all patients, patients younger than 50 years and patients aged 50 years or older

	**All patients (*n*=403) Mean (±s.d.) or percent *n***		**Age at diagnosis < 50 years (*n*=96) Mean (±s.d.) or percent *n***		**Age at diagnosis ⩾50 years (*n*=307) Mean (±s.d.) or percent *n***	
Age at diagnosis (years)	58.5 (11.5)	403/403	43.4 (5.2)	96/96	63.2 (8.4)	307/307
Age at menarche (years)	13.25 (1.3)	399/403	12.9 (1.2)	94/96	13.4 (1.3)	305/307
Pre-menopausal (%)	25	402/403	75	96/96	9	306/307
Age at menopause (years)	49.1 (5.1)	290/403	39.7 (5.8)	17/96	49.7 (4.4)	273/307
Pregnancies	2.3 (1.4)	403/403	2.5 (1.4)	96/96	2.2 (1.4)	307/307
Parity	1.9 (1.2)	403/403	1.8 (1.0)	96/96	1.9 (1.2)	307/307
Age at first full-term pregnancy, in parous women	25.1 (4.9)	334/403	26.6 (4.3)	82/96	24.6 (4.9)	252/307
OC	69	403/403	91	96/96	62	307/307
HRT, ever	45	403/403	11	96/96	56	307/307
Current smoker, yes	22	403/403	18	96/96	23	307/307
Current teetotaller, yes	11	403/403	13	96/96	11	307/307
1 and/or 2° relative with breast cancer	34	388/403	37	92/96	33	296/307
BMI (kg/m^2^)	25.3 (4.4)	401/403	24.3 (4.4)	96/96	25.6 (4.3)	305/307
Waist to hip ratio	0.84 (0.08)	399/403	0.82 (0.08)	94/96	0.84 (0.08)	305/307
Total breast volume (cm^3^)	1156 (682)	394/403	985 (574)	93/96	1208 (705)	301/307

Abbreviations: BMI, body mass index; HRT, hormone replacement therapy; OC, oral contraceptives.

**Table 2:** The allele frequencies of the *IGF1* CA-repeat allele in all patients. The percentage does not add up to 100%, because it was rounded off

	**All women (*n*=403)**	
**[CA]**_***n***_	**No. of alleles**	**%**
11	1	(0.1)
12	—	
13	—	
14	—	
15	1	(0.1)
16	—	
17	21	(2.6)
18	31	(3.8)
**19**	**525**	(**65.1)**
20	167	(20.7)
21	46	(5.7)
22	12	(1.5)
23	2	(0.2)
	*n*=806	

Abbreviations: CA, cytosine-adenine; IGF, insulin-like growth factor.

This is the most common repeat length.

The common 19 repeat allele is bolded.

**Table 3:** Age (mean,±s.d.) at breast cancer diagnosis in relation to multiparity and *IGF1* genotype

	**Age at breast cancer diagnosis**		
	***IGF1*+*19***	***IGF1-19/-19***	
0–1 children	58.0 (±12.1)	62.3 (±10.9)	*P*=0.23
	*n*=115	*n*=13	
2+ children	59.3 (±11.3)	53.1 (±9.5)	*P*=0.001
	*n*=236	*n*=39	
	*P*=0.33	*P*=0.006	*P_interaction_*=0.007

Abbreviations: CA, cytosine-adenine; IGF, insulin-like growth factor. There was a significant interaction on age at breast cancer diagnosis in multiparous patients and in patients having less then two children depending on the presence (*IGF1*+*19*) or absence (*IGF1-19/-19*) of the IGF1 19 CA-repeat allele
